# Evaluation of Structure and Assembly of Xyloglucan from Tamarind Seed (*Tamarindus indica* L.) with Atomic Force Microscopy

**DOI:** 10.1007/s11483-015-9395-2

**Published:** 2015-04-12

**Authors:** Arkadiusz Kozioł, Justyna Cybulska, Piotr M. Pieczywek, Artur Zdunek

**Affiliations:** Institute of Agrophysics, Polish Academy of Sciences, Doswiadczalna 4, 20-290 Lublin, Poland

**Keywords:** AFM, Xyloglucan, Nanostructure, Tamarind seeds

## Abstract

The role of xyloglucan (XG) in the cell wall of plants and its technological usability depends on several factors, pertaining to molecular structure. Therefore, the goal of this study was to evaluate the nano-structure and self-assembly of XG by atomic force microscopy (AFM). As the model, a non-modified xyloglucan from a tamarind seed (*Tamarindus indica* L.) was used. Samples were minimally processed, i.e., treated with low-power ultrasound and studied on the surface of mica in ambient butanol. AFM topographic images revealed rod-like nanomolecules of xyloglucan with a mean height of 2.3 ± 0.5 nm and mean length of 640 ± 360 nm. The AFM study also showed that XG chains possessed a helical structure with a period of 115.8 ± 29.2 nm. This study showed possible-bending of molecules with a mean angle of 127.8 ± 25.6°. The xyloglucan molecules were able to aggregate as cross-like and a parallel like assemblies, and possibly as rope-like structures. The self-assembled bundles of xyloglucan chains were often complexed at an angle of 114.2 ± 36.3°.

## Introduction

Xyloglucan (XG) is the most abundant polysaccharide from the group of hemicelluloses in the primary cell wall of higher plants comprising of about 20 % of all constituents of dicotyledons [1]. According to the dominating concept of architecture of a cell wall in plants, cellulose and hemicellulose construct a major load-bearing structure where XG has the structural function affecting orientation of cellulose microfibrils [2]. Together with pectic polysaccharides, xyloglucan is classified as a matrix polysaccharide. In cotyledon or some dicotyledon seeds it plays the role of a storage polysaccharide [3, 4] and may be present in the secondary cell wall. The nanostructure of a xyloglucan polysaccharide determines the cross-linking mechanism to cellulose microfibrils, i.e., a less substituted backbone of XG (fucosyl branching), the greater capability for binding to cellulose [5, 6]. The arrangement of XG chains contributes to a higher rigidity of the wall. This biopolymer is a heteropolysaccharide possessing the backbone composed of 1,4-β-D-glucose. In the backbone, three out of four glucose residues are substituted with 1,6-α-D xylose, which are about 70 % of glucosyl residues [7]. Additional residues, in form 1,2-β-D galactose, may be attached to xylose, depending on the origin of xyloglucan. Fucose can attach to outer galactose residue. A xyloglucan chain has a ribbon-like, twofold helical conformation [8, 9]. XG, as a cross-linking material, coats the surface of cellulose microfibrils, although some of them may penetrate and disrupt the microcrystalline parts of cellulose molecules [10].

Tamarind seeds are one of the most abundant sources of xyloglucan and commonly used as a storage of commercially available polysaccharides. Tamarind XG (like many other polysaccharides) is water-soluble, but its individual macromolecules tend not to fully hydrate, thus supramolecular aggregated chains remain present even in very diluted solutions [11]. The diversity of the possible side chains of a xyloglucan backbone determines the functionality and physico-chemical properties, like water solubility or gelling capability, therefore; currently tamarind xyloglucan is used e.g., as a gelling agent, an emulsion stabilizer or as a food thickener [4]. XG, like many other biopolymers, is potentially important in commercial and medical applications. Xyloglucan is also a source of biologically active oligosaccharides and was indicated as an antitumor factor [12, 13]. Lately, XG has been found as a material for the preparation of edible, non-toxic, biodegradable and transparent films for various applications especially in the controlled release of drugs and cosmetics [14–17].

Tamarind XG was examined by X-ray scattering and light scattering [11] to evaluate its structure or by chromatographic methods to estimate XG size and molecular weight [3, 18]. Most of the studies on tamarind xyloglucan also focused on the rheological or physico-chemical properties of chemically modified molecules [16, 19, 20]. Recently, AFM was used to study the structure of individual hemicelluloses extracted from the cell walls of fruit [21, 22]. So far, only a few experiments have been devoted to nanostructure characterization of natural, chemically non-treated, xyloglucan molecules or hemicellulose (understood as components of few hemicellulosic polysaccharides) from fruit [21, 23].

Thus, structural characterization is still indeed important since it determines the function of XG in plants and functionality for commercial use in food systems. In this paper, we would present the structural characterization of xyloglucan molecules with a particular emphasis on assemblies which could be formed by the XG molecules. For this purpose the XG molecules from tamarind seeds, as a model system, were studied with an atomic force microscope, where it is possible to obtain a nanometre resolution [24–29]. To study the structure of individual polysaccharides with AFM, molecules must be adsorbed on the mica [30–37]. Before placement on mica we applied weak ultrasound treatment and low dilution of an XG solution to obtain individual non-highly-aggregated molecules for an objective analysis. This method gave useful topographical information about the structure of XG rod-like molecules and their assemblies. Our experimental results on XG structure are in line with previous simulations of molecule dimensions by molecular dynamics.

## Materials and Methods

### Sample Preparation

Tamarind xyloglucan from seeds (*Tamarindus indica* L.) purchased from Megazyme (Bray, Ireland) was composed of xylose, glucose, galactose and arabinose in the proportion of 38:42:16:4. The 1 % (*w/v*) aqueous solution of the xyloglucan was diluted 20 times. Then it was stirred for 12 h in room temperature. Since XG has a tendency for self-association in aqueous solutions, the diluted sample (1.5 ml) was treated with low power ultrasounds (vibration amplitude 32 μm and power 130 W) for 30 s to reduce aggregates and macroparticles. The ultrasonic processor Vibra-Cell VCX130FSJ (Sonics & Materials, Inc., Newtown, USA) with microtip probe of 3 mm diameter was used. The treatment was optimized to possibly obtain a high yield of molecule deployment during AFM scanning.

For structure characterization, 5 μl of the xyloglucan solution was dropped onto the freshly cleaved mica and dried for 1 h in ambient air. This causes adhesion of molecules to substrate which facilitates scanning by AFM tip of biopolymers [21, 25, 38].

### AFM Imaging

Topographic observations were performed using the atomic force microscope Bioscope Catalyst II (Bruker, St. Barbara, U.S.A.) and semiautomatic PeakForce Tapping mode. The AFM imaging was carried out in butanol (POCH, Gliwice, Poland) which reduces undesirable effects appearing during imaging; decreases adhesion of the tip and reduces influence of the water layer on the surface [30, 37]. The height images were taken with resolution 512 × 512 points and 0.5 Hz scan rate with use of a silicon probe (Bruker) of the nominal spring constant 0.4 Nm^−1^ and the resonance frequency of 70 kHz. Measured RMS roughness on the mica surface at the same scanning conditions was 0.3 nm.

XG chains in this experiment were characterized by their length and height. Molecule height was chosen instead of width since for a typical AFM tip, the width of molecules is often even several dozen times bigger than their height due to so-called “profile broadening” or “tip convolution” effect [39]. Additionally, scanning in Z axis is more accurate than in X-Y due to scanner range size. In the case of chain length, this effect is not fundamentally important.

### Data Analysis

**Fig. 1:**
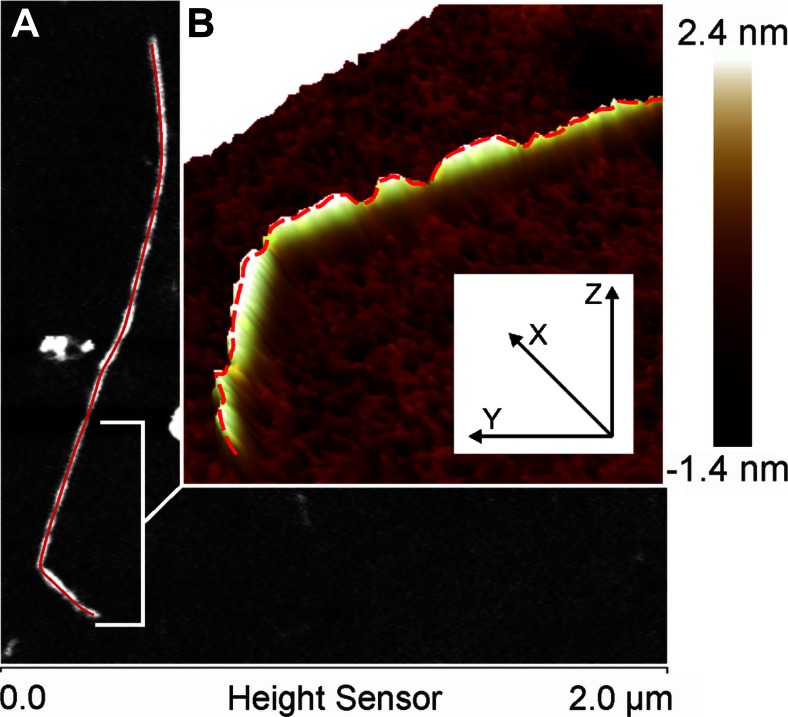
Method of determination of the height profile of chain-like molecules from an AFM height image. **a** The *red line* is a skeleton of XG molecule drawn from segmented images at a threshold 700 pm **b** The *dashed red line* depicts the height profile, which was determined along the skeleton. The height histogram was built from the each pixel along the line

The height images were subjected to the 3rd order flattening in SPIP 6.0.14 (Image Metrology, Hørsholm, Denmark) before quantitative analysis. Images were analyzed by self-developed protocol in Matlab (The MathWorks, MA, USA). In the image analysis protocol molecules were segmented from background. The minima threshold was set at 0.2 nm. Then a skeleton of the segmented objects was determined [22]. The skeleton was used as the mask to the original image to mark pixels where the heights along the molecules were collected (Fig. 1). The height histogram was built from each point defined by the skeleton. The skeletonized images were also used to evaluate the length of molecules. Angles and peak to peak distances were measured manually in computer image analysis SPIP 6.0.14 software. In the peak to peak analysis, the heights greater than 4 nm were considered to determine the distance between two adjacent peaks along the chain.

## Results and Discussion

**Fig. 2:**
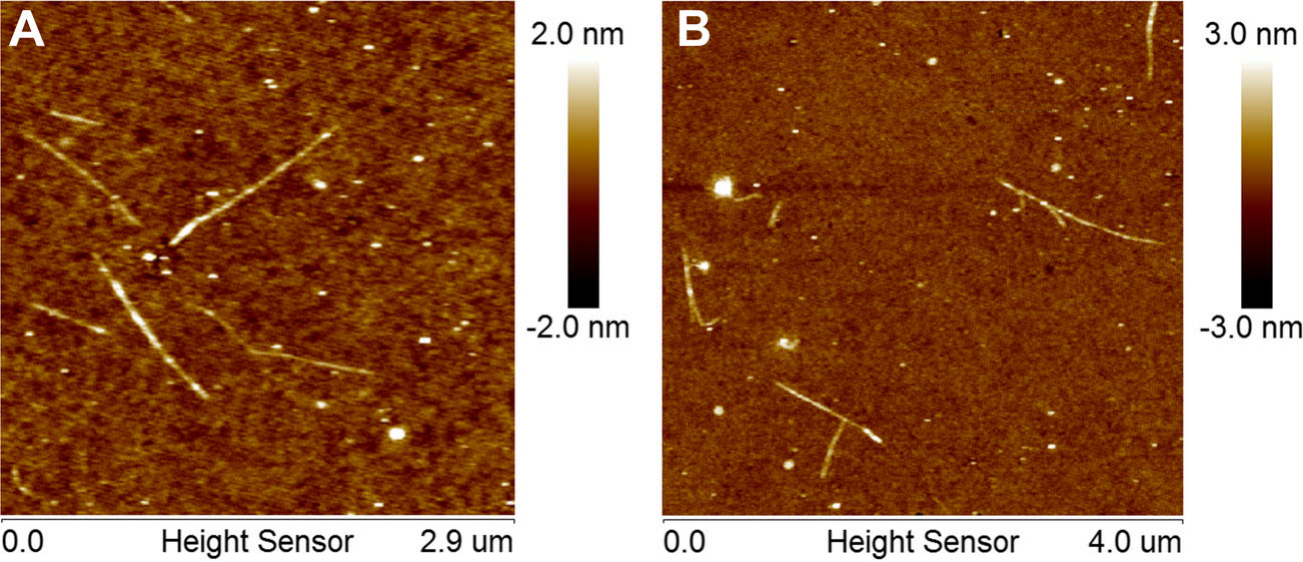
An overview on AFM height images of tamarind xyloglucan polysaccharide chains: individual (**a**), linked (**b**)

**Fig. 3:**
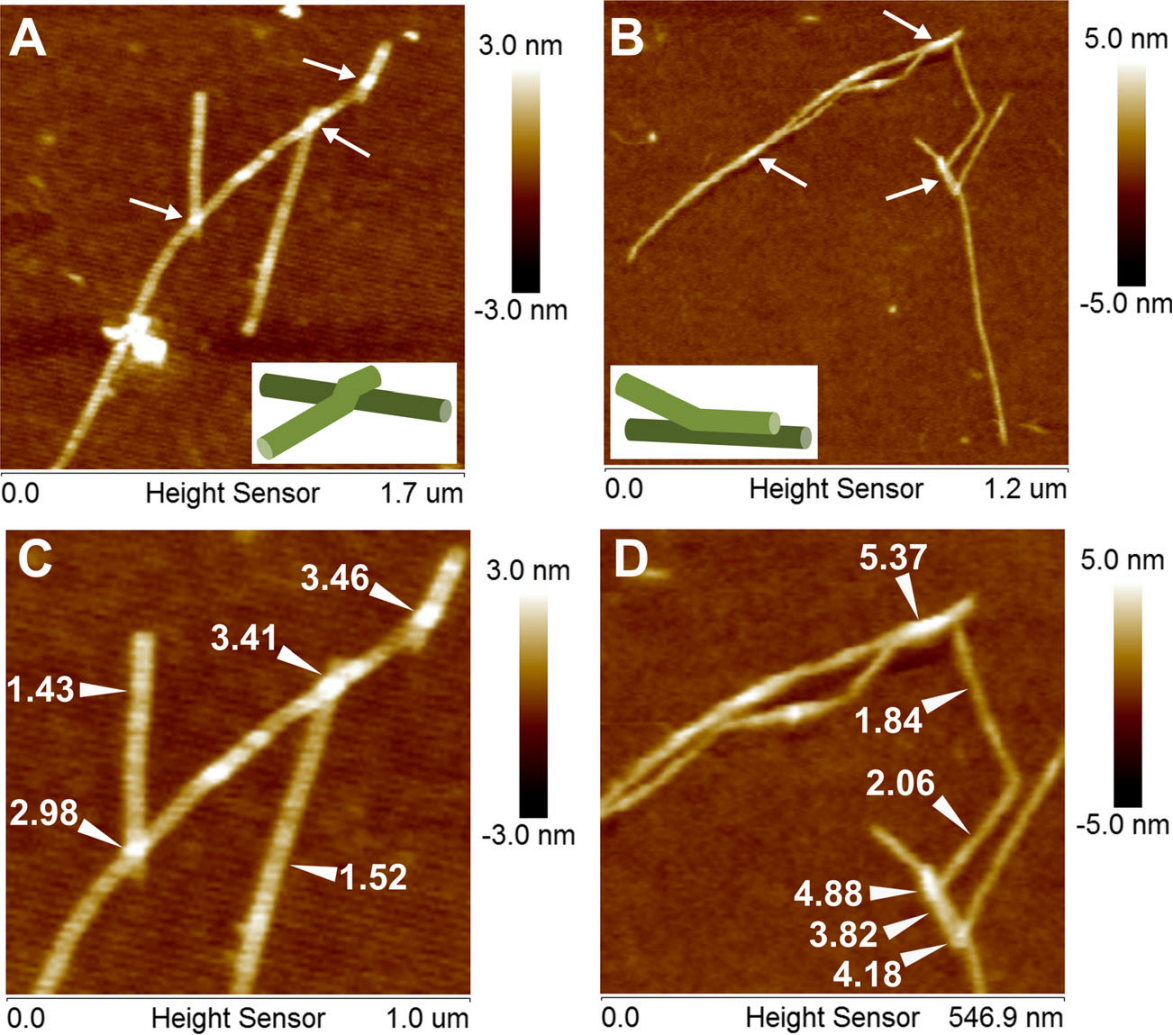
Examples of xyloglucan chains assemblies. **a** The connection of one end of a molecule to another with doubling the height at the linking point. **b** Assembly of a surface adherence. Adherence one chain parallel to each other causes increase of total height. Contact points or contact surface of chains are indicated by arrows. **c** and **d** present detailed analysis of the height (in nanometers) in the linking parts

Results of study are summarized in Table 1. AFM height images presented in Fig. 2 show that pure xyloglucan formed rectilinear, slender and rod-like chains. In the case of the presented AFM scans; the fibrils are deposited on a film-like matrix which covers the mica. A root mean square average of height deviations of mean data was about 0.6 nm, whereas; for clean mica it was about 0.3 nm. The film-like deposition on mica presumably consists of xyloglucan particles partially solubilized in water after ultrasound treatment. Consequently, the height of objects was determined relatively to the mean height of the background. The XG molecules were randomly spread as individual, separated rods (Fig. 2a) or as bundles of several chains (Figs. 2b and 3). Despite the ultrasound treatment priory to imaging XG molecules still showed a tendency for assembly. Two ways of linking between chains in the bundles were observed. The first way consisted in the linking of one end of the rod to another one in the middle of its chain (Fig. 3a). Profile analysis showed that this is rather an overlap of one molecule on another because the total height in the linking point was doubled (Fig. 3a and c). In the second way of assembly, chains were connected along some distance that also caused an increase of total height (Fig. 3b and d). The parallel connection of neighboring chains is likely to link the mechanism of cellulose by xyloglucan where a smooth XG region adheres by hydrogen bonds to cellulose. This result also confirms simulations performed on tamarind xyloglucan chains which showed the ability for parallel-arrangement [40]. The observation that xyloglucan molecules have a tendency to link is in line with AFM images of hemicelluloses obtained from Chinese cherries [21]. It suggests that xyloglucan in the cell wall of plants may form branched structures and in this way affects cell wall properties.

**Table 1:** Characterization of tamarind xyloglucan molecules

	Mean value	N_p_	N
Height (nm)	2.3 (0.5)^a^	6672	85
Length (nm)	640 (360)	85	85
Peak to peak distance (nm)	115.8 (29.2)	44	1 (the longest chain)
Angles			
Single molecules (deg)	127.8 (25.6)	19	16
Complexed molecules (deg)	114.2 (36.3)	14	10

N_p_ – number of data used for mean calculation

N – number of analyzed chains

^a^Standard deviation in parentheses

**Fig. 4:**
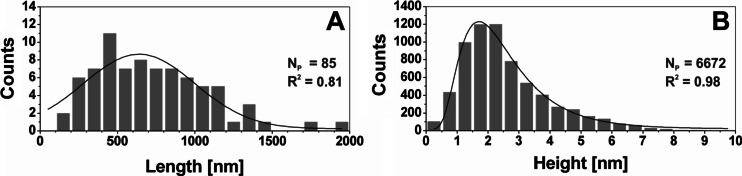
The distribution of the length **a** and the height **b** of xyloglucan molecules with fitting curves (Gaussian for the length, log-normal for the height). N_P_ means the number of data from histograms, non-branched XG chains taken for analyses (for length distribution it is a number of chains, for height distribution – number of height points along skeleton lines from all chains); R^2^ is coefficient of the determination of fitting. Polysaccharide chains longer than 2 μm have been omitted on graph **a** in order to show the essential part of the histogram

Image analysis allowed to characterize the dimensions of tamarind xyloglucan chains (Fig. 4). These characteristics were obtained for separated XG molecules and did not include the increase of height as the result of assembling. A length of tamarind xyloglucan chains was in a range from 178 nm up to 2 μm and the mean value from the fitting of normal distribution was 640 ± 360 nm (Fig. 4a). In a studied population of tamarind XG chains substantial fraction of molecules of the length ca. 300-1000 nm was noted. A previous study of xyloglucan from tamarind seeds showed that for trimer the length of spine was estimated as 3.54 nm (using gyration radii from SAXS experiment) [40]. Although the value was not exactly a multiplication of the monomer length it was concluded that one monomer unit adds approximately 1 nm to the total length of the spine. Taylor and Atkins [8] have analyzed the structure of xyloglucan polysaccharide from the tamarind seed. They have prepared a XG film and by using X-ray diffraction in order to show the length of XG’s repeating unit consisting of four D-glucose residues (XG monomer) was estimated as 2.06 nm. Small-angle neutron scattering showed the XG monomer persistence length as 8 nm [18]. It means that the mean length of tamarind XG molecules obtained in this experiment agrees with the theory that an XG backbone may contain from 300 to 3000 glucose units, which corresponds to the length from 0.15 to 1.5 μm, if each glucose unit is 0.5 nm [41].

**Fig. 5:**
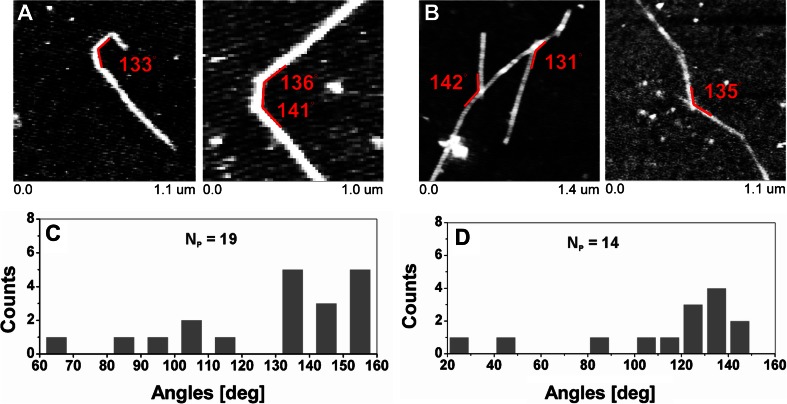
Characteristic angles of xyloglucan assemblies. **a** bending angle, **b** complexing angle, **c** distribution of bending angles, **d** distribution of the complexing angles. N_P_ is a number of events taken to analysis

The height distribution is presented in Fig. 4b. The height value of XG, which more accurately represents diameter due to the lack of the tip convolution effect, was within 0.5–7.0 nm. The mean height of XG from the log-normal distribution fitting was 2.3 ± 0.5 nm. A very similar diameter was found by an AFM study of hemicellulose extracted from a Chinese cherry [18]. For this material the chain width was 1.06–1.81 nm for ripe soft and 1.65–5.22 nm for ripe crisp fruit. Mkedder et al. [42] described tamarind xyloglucan nanoparticles prepared in NaNO_2_, deposited on hydrophilic silicon substrate and incubated with 1,4-β-endoglucanase enzyme that their diameter is from 5 to 12 nm. Molecular dynamic simulations estimated dimensions of XG monomers unit as 1.41-1.49 nm (length) and 0.62–0.75 nm (width) [40], depending on the number of side-chains. Muller et al. [20] determined the cross section, of a XG monomer as 0.63 nm. Kochumalayil et al. [43] estimated by molecular dynamics a simulation radius of XG monomer perpendicular to chain direction as 0.5 nm. Thus, the height of XG molecules observed in this experiment which was higher than 0.5 nm agrees with this result. Moreover, experimentally observed thickness may be affected by molecules bundling. In our study the single tamarind xyloglucan chains were linear with occasional bending (Fig. 5a). The most preferable bending angle was in a range of 130–160° (Fig. 5c) with a mean angle 127.8 ± 25.6°. The self-assembled bundles of xyloglucan chains were complexed at preferable angles in a range of 120-150° with a mean complexing angle of 114.2 ± 36.3° (Fig. 5b and d).

**Fig. 6:**
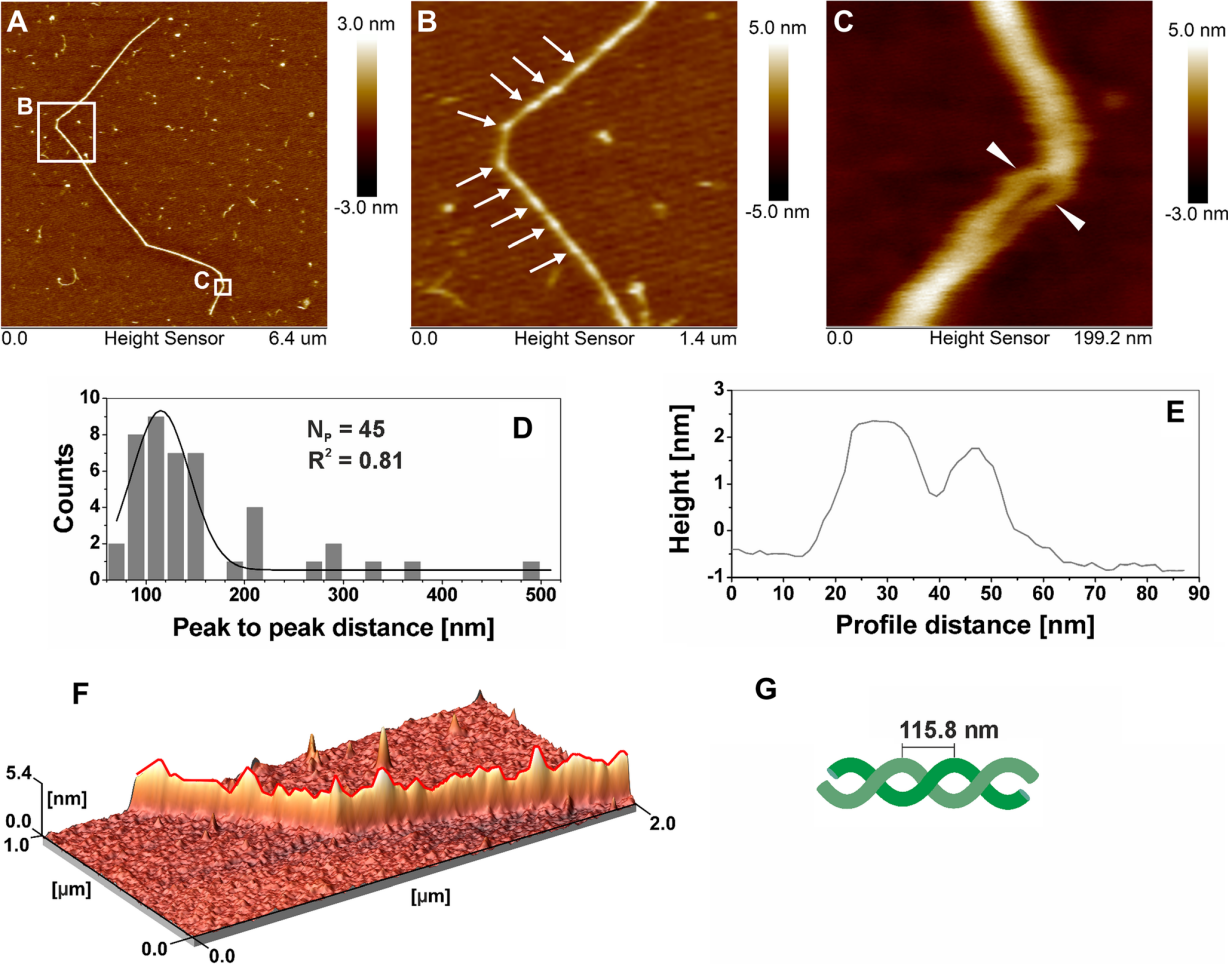
**a** Topographic image of a xyloglucan chain chosen for analysis of folding. **b** Periodicity on XG fiber. **c** The bending place with a split of chain in two chains. **d** Distribution of peak-to-peak on XG chains (N_P_ means the number of data from histograms, R^2^ is coefficient in the determination of fitting); **e** A profile of the case where a helical structure was untangled (marked in **c**). **f** 3D visualization of XG rope-like assembly; **g** Model of XG rope-like assembly with mean peak-to-peak distance

The folding of a xyloglucan chain is presented in Fig. 6. In this particular case, a single and very long chain of about 8.32 μm length was flexed in a few points (Fig. 6a). In Fig. 6b a periodic rope-like structure could be noted with peaks of 6 nm and hills 3 nm appearing with a mean period of 115.8 ± 29.2 nm (Fig. 6d). Detailed analysis revealed that this apparently single molecule was composed of at least two chains which could be discovered in Fig. 6c in the place where the chains were untangled. Figure 6e depicts the profile in this place. The tangling of two strains also explains a higher thickness of this molecule than the mean value (Fig. 6b). The three-dimensional image of the long folding chain (Fig. 6f) shows a periodic rope-like structure, which a simple model is presented in Fig. 6g. This rope-like assembly may be one of the possible linkage mechanisms due to twofold helical conformation of tamarind xyloglucan polymers [8].

Data obtained in this study shed new light on tamarind xyloglucan molecules. It may self-organize on three ways as described above. According to the literature, XG chains may interact not only with cellulose microfibers by hydrogen bonding, but also by covalent bonds, with pectin polysaccharides [44, 45], which consist of galacturonic acid residues and, among others neutral sugar residues, such as rhamnose, galactose or arabinose. So, different arrangements of XG molecules and structural features revealed in this work can be used for further modeling of cell wall assembly, as well eventually for the understanding of food and bioplastic systems.

## Conclusions

The AFM study showed that xyloglucan molecules from the tamarind seed have a rectilinear, slender and rod-like structure with a mean diameter of 2.3 ± 0.5 nm and mean length of 640 ± 360 nm. The AFM study also showed that XG chains possess a helical structure with a period of 115.8 ± 29.2 nm. The molecules may bend with a mean angle of 127.8 ± 25.6°. The xyloglucan molecules are able to aggregate as a cross-like at a preferential angle of about 114.2 ± 36.3° with parallel like assemblies, and probably as well as rope-like structures. The results have shown that AFM is a useful tool to unveil new features of a xyloglucan polysaccharide structure and its self-assembly.

## References

[1] Fry SC, Fry SC (1988). The Growing Plant Cell Wall: Chemical and Metabolic Analysis.

[2] Hayashi T (1989). Xyloglucans in the primary cell wall. Annu. Rev. Plant. Physiol. Plant. Mol. Biol..

[3] Freitas RA, Martin S, Santos GL, Valenga F, Buckeridge MS, Reicher F, Sierakowski M-R (2005). Physico-chemical properties of seed xyloglucans from different sources. Carbohydr. Polym..

[4] Nishinari K, Takemasa M, Zhang H, Takahashi R, Kamerling JP (2007). Comprehensive Glycoscience.

[5] Levy S, York WS, Stuike-Prill R, Meyer B, Staehelin LA (1991). Simulations of the static and dynamic molecular conformations of xyloglucan. The role of the fucosylated sidechain in surface-specific sidechain folding. Plant J..

[6] Lima DU, Loh W, Buckeridge MS (2004). Xyloglucan-cellulose interaction depends on the sidechains and molecular weight of xyloglucan. Plant Physiol. Biochem..

[7] Burton RA, Gidley MJ, Fincher GB (2010). Heterogeneity in the chemistry, structure and function of plant cell walls. Nat. Chem. Biol..

[8] Taylor IEP, Atkins EDT (1985). X-ray diffraction studies on the xyloglucan from tamarind (Tamarindus indica) seed. FEBS J..

[9] Umemura M, Yuguchi Y (2005). Conformational folding of xyloglucan side chains in aqueous solution from molecular dynamics simulation. Carbohydr. Res..

[10] Pauly M, Albersheim P, Darvill A, York WS (1999). Molecular domains of the cellulose/xyloglucan network in the cell walls of higher plants. Plant J..

[11] Picout DR, Ross-Murphy SB, Errington N, Harding SE (2003). Pressure cell assisted solubilization of xyloglucans: tamarind seed polysaccharide and detarium gum. Biomacromolecules.

[12] Cao Y, Ikeda I (2009). Antioxidant activity and antitumor activity (in vitro) of xyloglucan selenious ester and surfated xyloglucan. Int. J. Biol. Macromol..

[13] Kato Y, Uchida J, Ito S, Mitsuishi Y (2001). Structural analysis of the oligosaccharide units of xyloglucan and their effects on growth of COLO 201 human tumor cells. Int. Congr. Ser..

[14] Cerclier CV, Guyomard-Lack A, Cousin F, Jean B, Bonnin E, Cathala B, Moreau C (2013). Xyloglucan-cellulose nanocrystal multilayered films: effect of film architecture on enzymatic hydrolysis. Biomacromolecules.

[15] Chandroth KS, Tholath EA (2010). Biodegradable biocompatible xyloglucan films for various applications. Colloid Polym. Sci..

[16] Simi CK, Abraham TE (2009). Physico-chemical properties of aminated tamarind xyloglucan. Colloids Surf. B.

[17] Villares A, Moreau C, Capron I, Cathala B (2014). Chitin nanocrystal-xyloglucan multilayer thin films. Biomacromolecules.

[18] Muller F, Manet S, Jean B, Chambat G, Boué F, Heux L, Cousin F (2011). SANS measurement of semiflexible xyloglucan polysaccharide chains in water reveal their self avoiding statistics. Biomacromolecules.

[19] Sims IM, Gane AM, Dunstan D, Allan GC, Boger DV, Melton LD, Bacic A (1998). Rheological properties of xyloglucans from different plant species. Carbohydr. Polym..

[20] Pongsawatmanit R, Temsiriponga T, Ikeda S, Nishinari K (2006). Influence of tamarind seed xyloglucan on rheological properties and thermal stability of tapioca starch. J. Food Eng..

[21] Chen F, Zhang L, An H, Yang H, Sun X, Liu H, Yao Y, Li L (2009). The nanostructure of hemicellulose of crisp and soft Chinese cherry (Prunus pseudocerasus L.) cultivars at different stages of ripeness. LWT-Food. Sci. Technol..

[22] Zdunek A, Kozioł A, Pieczywek PM, Cybulska J (2014). Evaluation of the nanostructure of pectin. Hemicellulose and cellulose in the cell walls of pears of different texture and firmness. Food Bioprocess Technol..

[23] Jó TA, Petri DFS, Beltramini LM, Lucyszyn N, Sierakowski MR (2010). Xyloglucan nano-aggregates: Physico-chemical characterisation in buffer solution and potential application as a carrier for camptothecin, an anti-cancer drug. Carbohydr. Polym..

[24] Kirby AR (2011). Atomic force microscopy of plant cell walls. The plant cell wall. Methods Mol. Biol..

[25] Kirby AR, Gunning AP, Waldron KW, Morris VJ, Ng A (1996). Visualization of plant cell walls by atomic force microscopy. Biophys. J..

[26] Cybulska J, Konstankiewicz K, Zdunek A, Skrzypiec K (2010). Nanostructure of natural andmodel cellwallmaterials. Int. Agrophys..

[27] Cybulska J, Vanstreels E, Ho QT, Courtin CM, Van Craeyveld V, Nicolaï B, Zdunek A, Konstankiewicz K (2010). Mechanical characteristics of artificial cell walls. J. Food Eng..

[28] Cybulska J, Zdunek A, Konstankiewicz K (2011). Calcium effect on mechanical properties of model cell walls and apple tissue. J. Food Eng..

[29] Cybulska J, Zdunek A, Kozioł A (2015). The self-assembled network and physiological degradation of pectins in carrot cell walls. Food Hydrocoll..

[30] Kirby AR, Gunning AP, Morris VJ, Ridout MJ (1995). Observation of the helical structure of the bacterial polysaccharide acetan by atomic force microscopy. Biophys. J..

[31] Kirby AR, Gunning AP, Morris VJ (1995). Imaging xanthan gum by atomic force microscopy. Carbohydr. Res..

[32] Kirby AR, MacDougall AJ, Morris VJ (2008). Atomic force microscopy of tomato and sugar beet pectin molecules. Carbohydr. Polym..

[33] Liu H, Chen F, Yang H, Yao Y, Gong X, Xin Y, Ding C (2009). Effect of calcium treatment on nanostructure of chelate-soluble pectin and physicochemical and textural properties of apricot fruits. Food Res. Int..

[34] Yang H, An H, Feng G, Li Y, Lai S (2005). Atomic force microscopy of the water-soluble pectin of peaches during storage. Eur. Food Res. Technol..

[35] Zhang L, Chen F, Yang H, Sun X, Liu H, Gong X, Jiang C, Ding C (2010). Changes in firmness, pectin content and nanostructure of two crisp peach cultivars after storage. LWT- Food Sci. Technol..

[36] Zhang L, Chen F, Yang H, Ye X, Sun X, Liu D, Yang B, An H, Deng Y (2012). Effects of temperature and cultivar on nanostructural changes of water-soluble pectin and chelate-soluble pectin in peaches. Carbohydr. Polym..

[37] Pose S, Kirby AR, Mercado JA, Morris VJ, Quesada MA (2012). Structural characterization of cell wall pectin fractions in ripe strawberry fruits using AFM. Carbohydr. Polym..

[38] Foschiatti M, Hearshaw M, Cescutti P, Ravenscroft N, Rizzo R (2009). Conformational studies of the capsular polysaccharide produced by Neisseria meningitides group A. Carbohydr. Res..

[39] Gołek F, Mazur P, Ryszka Z, Zuber S (2014). AFM image artifacts. Appl. Surf. Sci..

[40] Urakawa H, Mimura M, Kajiwara K (2002). Diversity and versatility of plant seed xyloglucan. Trends Glycosci. Glycotechnol..

[41] Fry SC (1989). The structure and functions of xyloglucan. J. Exp. Bot..

[42] Mkedder I, Travelet C, Durand-Terrasson A, Halila S, Dubreuil F, Borsali R (2013). Preparation and enzymatic hydrolysis of nanoparticles made from single xyloglucan polysaccharide chain. Carbohydr. Polym..

[43] Kochumalayil JJ, Sehaqui H, Zhou Q, Berglund LA (2010). Tamarind seed xyloglucan – a thermostable high-performance biopolymer from non-food feedstock. J. Mater. Chem..

[44] Bauer WD, Talmadge KW, Keegstra K, Albersheim P (1973). The structure of plant cell walls. II. The hemicelluloses of suspension-cultured sycamore cells. Plant Physiol..

[45] Stevens BJH, Selvendran RR (1984). Structural features of cell wall polymers of the apple. Carbohydr. Res..

